# Prevalence of Type 2 Diabetes Mellitus Among Urban Bihari Communities in Dhaka, Bangladesh: A Cross-sectional Study in a Minor Ethnic Group

**DOI:** 10.7759/cureus.2116

**Published:** 2018-01-26

**Authors:** Muhammad Asaduzzaman, Shahanaz Chowdhury, Jahid Hossen Shahed, Mohammad Abdullah Heel Kafi, Md. Nazim Uzzaman, Mahfuza Talukder Flowra, MSA Mansur Ahmed

**Affiliations:** 1 Laboratory Sciences & Services Division, International Centre for Diarrhoeal Disease Research Bangladesh (icddr,b); 2 Department of Community Health, Bangladesh University of Health Sciences,dhaka,bangladesh; 3 Consultant, United Nations Children’s Fund, Dhaka, Bangladesh; 4 Infectious Diseases Division, International Centre for Diarrhoeal Disease Research Bangladesh (icddr,b); 5 Technical Training Unit, International Centre for Diarrhoeal Disease Research, Bangladesh (Icddr,B); 6 Faculty of Medicine, Universitas Gadjah Mada,indonesia; 7 Department of Community Medicine, Bangladesh University of Health Sciences,dhaka,bangladesh

**Keywords:** diabetes, bihari community, ethnicity, urban, bangladesh

## Abstract

Introduction

The prevalence, disease progression, and treatment outcomes for patients with type 2 diabetes vary significantly between ethnic groups. The Bihari community constitutes one of the most vulnerable populations in Bangladesh on the basis of access to health services and other fundamental rights. Our study aimed at finding out the prevalence and risk factors of type 2 diabetes among the Bihari adults in Dhaka city.

Methods

This cross-sectional community-based study was carried out among stranded Pakistanis (known as Bihari) living in camps in the Mirpur area from July 2014 to June 2015. Laboratory-based oral glucose tolerance test (OGTT) was the basis for the diagnosis of type 2 diabetes mellitus (DM). Anthropometric measurements, blood pressure, biochemical tests, family history, and socioeconomic information were obtained to determine the risk factors.

Results

The prevalence of diabetes mellitus (DM), impaired glucose tolerance (IGT), and impaired fasting glucose (IFG) were estimated at 10.11%, 8.74%, and 4.55%, respectively. Increased diastolic blood pressure, serum triglyceride, and cholesterol level were observed to be significantly (p < 0.05) associated with diabetes. Also, the presence of diabetes, high blood pressure, and obesity among relatives significantly increased the probability of diabetes.

Conclusions

To the best of our knowledge, this is the first study on diabetes prevalence among the Bihari community in Bangladesh. The prevalence of type 2 diabetes mellitus was found to be higher among the Bihari community compared to the general population in Bangladesh. Health planners and policymakers should realize the alarming situation and identified risk factors and consider the minor ethnic groups during decision-making regarding prevention and control of diabetes and other noncommunicable diseases.

## Introduction

Diabetes is one of the four major types of noncommunicable diseases (NCDs) that make the largest contribution to morbidity and mortality worldwide [1]. In 2016, about 422 million people globally had diabetes, with most living in the developing countries, and unfortunately, more than 80% of diabetes deaths occur in low- and middle-income countries [2]. The prevalence of diabetes is increasing in Bangladesh in both urban and rural areas. A recent scoping review (1994-2013) revealed that the prevalence of type 2 diabetes varied from 4.5% to 35.0% in Bangladesh [3]. The overall age-adjusted (≥ 35 years) prevalence of diabetes was estimated as 9.7%, based on Bangladesh Demographic and Health Survey (BDHS) 2011 data [4].

A recent population-based study on the Santhal tribe (an ethnic group) of Bangladesh published in 2014 revealed that the prevalence of type 2 diabetes mellitus (DM) was only 0.6% and hyperglycemia (fasting plasma glucose > 5.5 mmol/l) was 10% [5]. The reason behind these insulin resistance phenomena is not straightforward. Multidimensional risk factors are associated with type 2 diabetes, namely modifiable and non-modifiable risk factors like obesity, physical inactivity, sedentary lifestyle, and gender, age, environmental influence etc., respectively. There is evidence to suggest that the prevalence, disease progression, and treatment outcomes for people with type 2 diabetes vary significantly between ethnic groups [6].

The Bihari people in Bangladesh, also known as non-Bangladeshi or stranded Pakistanis, are an ethnic minority, and at present over 250,000 Biharis live in 66 squalid refugee camps across the country [7]. They are still culturally isolated and speak their native Urdu. The slum-like camps are overcrowded with a very negligible population (less than 5%) having formal education [8]. The vulnerability of this community regarding access to essential healthcare services and other fundamental rights may force them to adopt different ecological conditions, which is reflected through potential differences in socio-economic and demographic condition. To our knowledge, no study has been conducted to describe diabetes among the Bihari communities in Bangladesh. Our study aimed at finding out the prevalence and the risk factors of type 2 diabetes among the Bihari adults (≥ 30 years) in Dhaka city.

## Materials and methods

Study design

This was a cross-sectional community-based study carried out from July 2014 to June 2015.

Study settings, sample size, and study population

The study was carried out among the Bihari communities living in the Mirpur area of Dhaka North City Corporation (DNCC). Apparently healthy adult volunteers of the seven camps (named as Talat camp, Millat camp, Consol camp, Irani camp, Kashmiri camp, Madrasa camp, and Rahamat camp) of the study area were randomly selected as study participants.

The calculated sample size was 284 by considering the prevalence rate at 6.1% of type 2 diabetes mellitus among the general population of Bangladesh [9] and error= 0.057. As the study population were camp dwellers with very low or no chance of migration, we considered < 1% drop out, and therefore we included 286 participants in our study.

Inclusion Criteria

People (both males and females) aged ≥ 30 years who agreed to participate voluntarily were selected as study participants.

Exclusion Criteria

Subjects aged < 30 years and not willing to participate were excluded from the study.

Data collection procedure

Four trained data collectors were assigned to prepare a list of eligible subjects (≥ 30 years) of the selected camps. After random selection of the participants from the camp dwellers’ list, written informed consents were taken from each individual and they were given a study-specific identification number and card. Face-to-face interviews were conducted using pretested semi-structured questionnaires, and each interview session took around 45 minutes. Nutritional status was assessed by anthropometric measurement of weight, height, hip and waist circumference. Basic information such as age, sex, educational status, occupation, family income, food intake pattern, smoking and alcohol consumption history as well as information about the various factors that affect the blood glucose level were also collected. During the interview, the participants were requested to provide a sample for measuring their blood glucose level using the oral glucose tolerance test (OGTT) and lipid profile on a specific date with clear instruction. All tests were performed in the biochemistry laboratory of the Bangladesh Institute of Health Sciences (BIHS) and the study participants were informed about the test results. The data were checked meticulously after collection to avoid errors or inconsistencies.

Data analysis

Data analysis was performed using Stata 13 (StataCorp LP, Texas, USA). The prevalence rate of diabetes was determined by percentage. Values were reported as the mean ± standard deviation (SD). Statistical comparisons between different groups were made using the t-test and χ² test. Logistic regression was applied to calculate the crude odds ratio and adjusted odds ratio to identify risk factors. Stepwise model selection using the Akaike information criterion (SWAIC model) [10] was used to get the significant risk factors. A p-value of < 0.05 was considered statistically significant.

Operational definitions

The World Health Organization (WHO) 2006 criteria [11] were followed to measure the parameters.

Diabetes Mellitus (DM)

It has been determined by measuring the venous plasma glucose level by OGTT at 0 minute and 120 minutes. If the 0 minute glucose level is ≥ 7.0 mmol/l and the 120 minute glucose level is ≥ 11.1 mmol/l, the person is considered as diabetic.

Impaired Glucose Tolerance (IGT)

It has been determined if the 0 minute glucose level is < 7.0 mmol/l and the 120 minute glucose level is between 7.8 to < 11.1 mmol/l.

Impaired Fasting Glucose (IFG)

When the 0 minute glucose level is 6.1 to < 7.0 mmol/l and the 120 minute glucose level is < 7.8 mmol/l.

Normal Glucose Tolerance (NGT)

If the 0 minute glucose level is < 6.1 mmol/l and 120 minute glucose level is < 7.8 mmol/l.

Ethnicity

A complex multidimensional construct reflecting the confluence of the biological factors and geographical origins, culture, economic, political and legal factors, as well as racism [12].

Anthropometric Assessment

The measurement of the physical dimension and the gross composition of the body. In this study, the body mass index (BMI) and waist-hip ratio (WHR) have been used.

Body Mass Index (BMI)

It is calculated by the formula: weight in kilogram divided by height in meter squared. It helps to grade the obesity. A BMI ≥ 25 is graded as overweight.

Hypertension (HTN)

Defined as a systolic blood pressure of ≥ 140 mm Hg and/or diastolic blood pressure of ≥ 90 mm Hg.

Lipid Profile

Based on the criteria described by ES Ford et al. [13].

Hypercholesterolemia

Fasting venous blood cholesterol level ≥ 200 mg/dl.

Triglycerides Level

Fasting venous blood triglycerides level considered abnormal if it is > 150 mg/dl.

Total Monthly Income

It includes pay and allowances drawn by the respondent plus any income from other family members.

Vigorous Intensity Physical Activities

Activities that require hard physical effort and cause large increases in respiratory or heart rate.

Moderate Intensity Physical Activities

Activities that require moderate physical effort and small increases in respiratory or heart-rate.

Sedentary/Mild Intensity Physical Activities

Sitting or reclining at work, at home, getting to and from places, or with friends, including time spent sitting at the desk, sitting with friends, traveling in the car, bus, train, reading, playing cards, or watching television, but not including time spent sleeping.

## Results

Sociodemographic information

The mean age of the male subjects was 47.7 years and that of the female participants was 41.8 years, whereas about 41% of the total study participants were between 30-39 years of age. There were no formal years of schooling among 85% of the participants. The types of occupation varied among the participants. Most of the male respondents (60%) were involved in business, whereas the females were mostly (61%) engaged in household activities. Monthly income ranged between Bangladeshi Taka (BDT) 5001 to 10,000 for more than half of the respondents. However, monthly income ≤ 5000 BDT constituted about 30% of the study population, which indicates low socioeconomic status (Table 1).

**Table 1:** Distribution of the respondents according to demographic characteristics. 82.80 Bangladeshi Taka (BDT) = 1 United States Dollars (USD).

Variables		Percentage		Percentage	Total	Percentage
Sex	Male		Female			
	N = 65	22.73	N = 221	77.27	N = 286	
Age groups in years						
30 – 39	17	26.15	99	44.80	116	40.56
40 – 49	18	27.69	75	33.94	93	32.52
50 – 59	19	29.23	24	10.86	43	15.03
≥ 60	11	16.92	23	10.41	34	11.89
Mean age	47.7		41.8			
Education						
Illiterate	54	83.07	188	85.07	242	84.62
Primary	6	9.23	22	9.95	28	9.79
Secondary	2	3.08	8	3.62	10	3.50
Higher secondary	3	4.62	3	1.36	6	2.10
Occupation						
Domestic work	2	3.08	135	61.09	137	47.90
Business	39	60.00	57	25.79	96	33.57
Service holder	10	15.38	3	1.36	13	4.55
Day laborer	6	9.23	0	0	6	2.10
Others	8	12.31	26	11.76	34	11.89
Monthly income (BDT)						
≤ 5000	18	27.7	67	30.3	85	29.7
5001-10,000	36	55.4	126	57	162	56.6
10,001-15,000	7	10.8	20	9	27	9.4
15,001-20,000	4	6.2	6	2.7	10	3.5
> 20,000	0	0	2	0.9	2	0.7

Lifestyle, family history, clinical, and anthropometric measurements

Most of the participants (76%) were engaged in moderate physical activities. Smoking habit among the males and females was 71% and 32%, respectively. Around 20% of the male respondents used to consume alcohol (Table 2). Almost 50% of the enrolled participants had a positive family history of DM, of which first (1st) and second (2nd) degree relatives account for 36% and 14%, respectively (Table 3). The BMI was found to be significantly (p-value <0.05) higher among the female participants compared to the male participants (Table 4).

**Table 2:** Distribution of the respondents according to lifestyle or behavioral characteristics.

Characteristics	Male	Percentage	Female	Percentage	Total	Percentage
	N = 65	22.73	N = 221	77.27	N = 286	
Physical activities						
Vigorous intensity	4	6.15	0	0	4	1.40
Moderate intensity	47	72.30	169	76.48	216	75.52
Vigorous + moderate intensity	7	10.77	14	6.33	21	7.34
Mild intensity	7	10.77	38	17.19	45	15.74
Smoking habit						
Yes	46	70.77	71	32.13	117	40.91
No	19	29.23	150	67.87	169	59.09
Alcohol consumption						
Yes	13	20	1	0.45	14	4.90
No	52	80	220	99.55	272	95.10

**Table 3:** Distribution of the respondents according to family history.

Variables		Percentage		Percentage		Percentage
Sex	Male		Female		Total	
	N = 65	22.73	N = 221	77.27	N = 286	
Presence of diabetes mellitus among relatives						
1^st^ degree relatives	9	13.85	94	42.53	103	36.01
2^nd ^degree relatives	6	9.23	33	14.93	39	13.64
No	33	50.77	30	13.57	63	22.03
Does not know	17	26.15	64	28.96	81	28.32
Presence of relative having high blood pressure						
1^st^ degree	14	21.54	36	16.29	50	17.48
2^nd^ degree	13	20	38	17.19	51	17.83
No	25	38.46	90	40.72	115	40.21
Does not know	13	20	57	25.79	70	24.48
Presence of relative who has obesity						
1^st^ degree	7	10.77	37	16.74	44	15.38
2^nd^ degree	5	7.69	28	12.67	33	11.54
No	48	73.85	142	64.25	190	66.43
Does not know	5	7.69	14	6.33	19	6.64

**Table 4:** Distribution of the respondents according to mean values (standard deviation (SD) for anthropometric and clinical variables.

Variables			P value
Sex	Male (N = 65)	Female (N = 221)	
	Mean (SD)	Mean (SD)	
Anthropometric variables			
Height (cm)	160.49 (7.30)	148.70 (8.68)	0.000
Weight (kg)	57.91 (11.44)	56.73 (13.49)	0.477
Waist circumference (cm)	81.53 (12.16)	88.24 (25.31)	0.039
Body mass index (BMI)	22.50 (4.26)	25.67 (5.15)	0.000
Waist to hip ratio (WHR)	0.92 (0.13)	0.93 (0.11)	0.653
Clinical variables			
Systolic blood pressure (mm Hg)	114.10 (22.74)	112.89 (21.82)	0.698
Diastolic blood pressure (mm Hg)	71.46 (10.61)	72.56 (11.39)	0.489
Fasting blood glucose (FBG) mmol/l	4.75 (0.19)	5.28 (0.19)	0.165
Oral glucose tolerance test (OGTT) mmol/l	6.33 (0.33)	7.26 (0.29)	0.108
Cholesterol	127.67 (4.75)	139.51 (3.05)	0.057
Triglyceride	125.23 (11.70)	138.01 (5.60)	0.292

Prevalence of type 2 diabetes

The overall prevalence of type 2 diabetes, impaired glucose tolerance, and impaired fasting glucose was estimated at 10.11%, 8.74%, and 4.55%, respectively. Increased diastolic blood pressure, serum triglyceride, and cholesterol level were observed to be significantly (p-value < 0.05) associated with diabetes (Table 5).

**Table 5:** Distribution of overweight, hypertension, and hyperlipidemia among the respondents in different categories of glucose tolerance. Abbreviations- SBP: systolic blood pressure; DBP: diastolic blood pressure; IFG: impaired fasting glucose; IGT: impaired glucose tolerance; DM: diabetes mellitus.

Variables	NGT	P-value	IFG	P-value	IGT	P-value	DM	P-value
	No. (%)		No. (%)		No. (%)		No. (%)	
Total respondents	221 (77.27)	0.204	13 (4.55)	0.518	25 (8.74)	0.874	29 (10.11)	0.093
Male	54 (18.88)		2 (0.70)		6 (2.10)		3 (1.05)	
Female	167 (58.39)		11 (3.85)		19 (6.64)		26 (9.09)	
Raised body mass index	102 (35.66)	0.662	8 (2.80)	0.277	8 (2.80)	0.119	17 (5.94)	0.18
Increased waist to hip ratio	34 (11.89)	0.243	3 (1.05)	0.534	6 (2.10)	0.312	5 (1.75)	0.944
High systolic blood pressure	17 (5.94)	0.129	2 (0.70)	0.419	3 (1.05)	0.596	4 (1.40)	0.353
High diastolic blood pressure	0	0.065	0	0.827	0	0.757	1 (0.35)	0.003
Increased triglyceride	53 (18.53)	0	9 (3.15)	0.002	7 (2.45)	0.813	19 (6.64)	0
Hypercholesterolemia	26 (9.09)	0	6 (2.10)	0.003	2 (0.70)	0.249	13 (4.55)	0

Risk factors for diabetes

The crude and adjusted odds ratio for factors affecting diabetes was determined by applying logistic regression. When the risk factors were calculated individually based on the DM positivity, no risk factor was recorded to be statistically significant, which meant that there might have been the confounding effect of other factors. However, when all the risk factors obtained from the current study were adjusted by the respondent’s smoking habit, drinking habit, the presence of high blood pressure (BP), moderate intensity activity, and use of salt in regular meals, it was observed that the presence of relatives with diabetes, high blood pressure, and obesity significantly affected the probability of type 2 diabetes (Table 6).

**Table 6:** Distribution of the different risk factors among the respondents. Observed as significant risk factors for diabetes mellitus (DM) after stepwise model selection using Akaike information criterion [10].

Risk factors	Crude			Adjusted		
	Odds ratio	P-value	Confidence interval	Odds ratio	P- value	Confidence interval
Relative with diabetes mellitus	1.558	0.098	0.922, 2.637	2.16	0.027	1.091, 4.277
Relative with high blood pressure	0.659	0.149	0.374, 1.161	0.39	0.025	0.174, 0.891
Relative with obesity	1.109	0.705	0.648, 1.898	2.23	0.030	1.081, 4.608
Smoking	0.871	0.731	0.395, 1.918	2.24	0.127	0.794, 6.323
Involved in moderate activity	0.614	0.295	0.246, 1.529	0.78	0.708	0.223, 2.767
Previous history of high blood pressure	1.498	0.383	0.604, 3.721	1.61	0.424	0.497, 5.267
Consumption of alcohol	1.003	0.995	0.394, 2.554	1.31	0.489	0.607, 2.836
Regularly using salt to meals	0.942	0.875	0.445, 1.992	1.02	0.971	0.364, 2.853

## Discussion

Differences in the prevalence, risk factors, and other adverse health conditions among specific population groups exist in type 2 diabetes mellitus throughout the world. There are multiple factors that contribute to these disparities, including biological, clinical, health system, and social factors [14]. Our study findings regarding type 2 diabetes prevalence and the risk factors among the Bihari population in Bangladesh have resemblance with similar studies [15]. In our study, the prevalence of diabetes was estimated higher in the 40-49 years age group in males and 50-59 years age group in females, which is consistent with previous study findings for developing countries that most people with diabetes are aged between 45 and 64 years [16]. Population below 45 years of age may be included due to distribution of the study population.

There is an inverse association between socioeconomic status and the prevalence of type 2 diabetes in the middle years of life [17]. A study conducted in the United States of America revealed that diabetes was over twice as prevalent among African-Americans (10.3%) when compared to Whites (4.6%; odds ratio (OR) = 2.38; 95% confidence interval (95% CI): 1.50, 3.75; P = 0.0001). This finding suggests that exposure to factors that contribute to the causation of diabetes is more common in deprived areas, as well as has racial difference [18].

Most studies reported a twofold to sixfold increased risk of type 2 diabetes associated with a positive family history compared to a negative family history of diabetes. These estimates are consistently elevated across different study designs and in several ethnic groups [19]. Our study also supports these data and exhibits a significant relationship between diabetes and positive family history of DM, hypertension, and obesity. Relevant nutritional measurements like BMI, waist to hip ratio (WHR) taken from the study population were found very much related to the causation of DM. The American Diabetes Association (ADA) recognizes BMI ≥ 25 kg/m2 as a risk factor for type 2 diabetes [20]. In our study, 102 subjects (35%) were overweight. Among the overweight population, 33 subjects (32%) had abnormal glucose metabolism, which includes 17 diabetic, eight IGT, and eight IFG cases. Therefore, among the diabetic cases, 58% had BMI ≥ 25 kg/m2. Two national surveys conducted by Bays et al. [21] revealed that increased BMI was associated with the increased prevalence of diabetes mellitus, hypertension, and dyslipidemia, and more than 75% of the patients had BMI ≥ 25 kg/ m2. Although, the BMI rate was found a bit lower, which may be due to the lesser sample size and differences in the background information of the participants of the two studies. Furthermore, 48 participants (17%) had an abnormal waist-hip ratio, of which 29% was suffering from either DM or glucose intolerance, which reinforces the previous findings that increased WHR is closely associated with increased risk of type 2 diabetes [22].

Minorities commonly live in substandard neighborhood environments with the lack of healthy food stores, lack of places to exercise, and increased psychosocial stressors related to crime or limited social cohesion that results in poor health outcomes [23-24]. Evidence from the multiethnic study of atherosclerosis found that inferior neighborhoods were associated with increased smoking, physical inactivity, and poorer control of blood pressure, which can contribute to the development of diabetes and its complications [25]. More than 30% of our study subjects were found to have an increased serum triglyceride level, out of which 35 subjects (40%) had abnormal glucose metabolism. It again established the link between glucose and lipid metabolism. But hypercholesterolemia was less evident both in the diabetic and normoglycaemic subjects. Only 47 (16%) people had a higher cholesterol level, of which 13 participants were diabetic. Hypertriglyceridemia, reduced high-density lipoprotein (HDL), and increased low-density lipoprotein (LDL) particles characterize dyslipidemia, which is a risk factor for coronary artery disease (CAD), dyslipidemia, hypertension, stroke, and type 2 diabetes [26].

About 41% of the respondents in our study were known to be smokers. It was alarming that 32% female participants gave the history of tobacco consumption. Although we did not find a significant association between smoking and diabetes, previous study findings showed that diabetes incidence was higher among smokers who used to smoke cigarettes ≥ two packs/day compared to those who had never smoked [27]. Considering the social and religious aspect of the study population in Bangladesh, the alcohol consumption seems to be high among the respondents (5% of the total participants). Alcohol intake was found to be associated with the risk of non-insulin-dependent diabetes mellitus, especially in men [28]. Numerous studies have found a contrary relationship between type 2 diabetes and education, occupation, and income that is consistent across all adult age groups. In our study, the literacy rate was found to be very poor among Bihari communities where 85% of the total population had no formal education. More than a quarter of the study participants had a monthly income ≤ 5000 BDT. Robbins et al. found that diabetes prevalence is more strongly associated with poverty [29]. Health care access and health insurance are important factors that allow patients with diabetes to receive adequate medical care.

The findings of this study may not be generalizable to all ethnic communities or the whole Bihari population in Bangladesh as the data was collected only from Dhaka city. Therefore, the prevalence and risk factors for diabetes might not be similar among the other Bihari camp residents in different parts of the country. As self-reported information was collected from the patients, there might be a chance of interviewer bias and recall bias. Other limitations include small sample size, cross-sectional study design, and the lack of follow-up, though we made suggestions to the respondents with positive findings to consult with clinicians.

## Conclusions

The Bihari community constitutes one of the most vulnerable populations in Bangladesh on the basis of access to health care services and other fundamental rights. This study reveals the high prevalence of type 2 diabetes mellitus among the Bihari communities compared to the general population. Health planners and policymakers should realize the alarming situation and identified risk factors and should consider the minority ethnic groups during decision-making regarding prevention and control of diabetes and other noncommunicable diseases.
